# Skin Autofluorescence, a Non-Invasive Marker for AGE Accumulation, Is Associated with the Degree of Atherosclerosis

**DOI:** 10.1371/journal.pone.0083084

**Published:** 2013-12-23

**Authors:** Martijn A. M. den Dekker, Marjan Zwiers, Edwin R. van den Heuvel, Lisanne C. de Vos, Andries J. Smit, Clark J. Zeebregts, Matthijs Oudkerk, Rozemarijn Vliegenthart, Joop D. Lefrandt, Douwe J. Mulder

**Affiliations:** 1 Center for Medical Imaging – North East Netherlands, University of Groningen, University Medical Center Groningen, Groningen, The Netherlands; 2 Department of Radiology, University of Groningen, University Medical Center Groningen, Groningen, The Netherlands; 3 Department of Internal Medicine, Division of Vascular Medicine, University of Groningen, University Medical Center Groningen, Groningen, The Netherlands; 4 Department of Epidemiology, University of Groningen, University Medical Center Groningen, Groningen, The Netherlands; 5 Department of Surgery, Division of Vascular Surgery, University of Groningen, University Medical Center Groningen, Groningen, The Netherlands; Brigham and Women’s Hospital, Harvard Medical School, United States of America

## Abstract

**Introduction:**

Advanced glycation endproducts (AGEs) may be involved in the development of atherosclerosis, beyond diabetes and renal disease. Skin autofluorescence (AF) is a non-invasive marker for AGEs. We examined whether skin AF is increased in (subclinical) atherosclerosis and associated with the degree of atherosclerosis independent of diabetes and renal function.

**Methods:**

A cross-sectional study of 223 patients referred for primary (*n* = 163) or secondary (*n* = 60) prevention between 2006 and 2012 was performed. Skin AF was measured using the AGE-Reader. Ultrasonography was used to assess plaques in carotid and femoral arteries and computed tomography for the calculation of the coronary artery calcium score (CACS; in primary prevention only). Primary prevention patients were divided into a group with subclinical atherosclerosis defined as >1 plaque or CACS>100 (*n* = 67; age 53 year [interquartile range 48–56]; 49% male) and without (controls; 96; 43 [38–51]; 55%). Secondary prevention were patients with peripheral arterial disease (60; 64 [58–70]; 73%).

**Results:**

Skin AF was higher in subclinical and clinical atherosclerosis compared with controls (skin AF 2.11 [interquartile range 1.83–2.46] and 2.71 [2.15–3.27] vs. 1.87 [1.68–2.12] respectively; *P* = 0.005 and <0.001). In a multivariate analysis, the association of skin AF with the atherosclerosis categories was independent of age, sex, diabetes, presence of the metabolic syndrome, Framingham Risk Score, and renal function. Skin AF correlated with most cardiovascular risk factors, Framingham risk score, and IMT and CACS.

**Conclusions:**

Skin AF is increased in documented subclinical and clinical atherosclerosis, independent of known risk factors such as diabetes and renal disease. These data suggest that AGEs may be associated with the burden of atherosclerosis and warrant a prospective study to investigate its clinical usability as a risk assessment tool for primary prevention.

## Introduction

Atherosclerosis is characterized by chronic low grade inflammation and oxidative stress leading to plaque formation and ultimately calcification. [1] While formerly only implicated in diabetes and renal disease, evidence for an important role of advanced glycation endproducts (AGEs) in cardiovascular disease (CVD) beyond these conditions is growing. [2] AGEs are formed by non-enzymatic glycation and oxidative reactions leading to stable structures accumulating on long-lived proteins. They promote cellular stress responses by engagement of the receptor for AGEs (RAGE). AGE epitopes have been detected in human plaques. [3] Lowering AGEs or blocking RAGE in murine models has been found to attenuate plaque formation, supporting the involvement of AGEs in atherosclerosis [3], [4].

Measurement of tissue AGEs may be preferable over plasma measurement, since long-lived proteins accumulate in the tissues in which chronic complications develop. [5] Thus, blood and urine AGEs do not necessarily reflect their tissue levels. [6] We developed and validated a non-invasive technique to quantify tissue AGEs by measuring skin autofluorescence (AF). [7], [8] It has been validated with skin biopsies in patients with diabetes or renal disease and healthy controls [7]–[9] and was shown to correlate strongly with plasma circulating AGEs and with corneal and lens fluorescence in type 1 diabetes. [10] Skin AF is elevated in diabetes mellitus and end-stage renal disease and is associated with cardiovascular mortality, independent of known CVD risk factors. [8], [11] Skin AF is also elevated in coronary artery disease, [12], [13] correlates with carotid intima media thickness (IMT), [14] and is elevated in patients with carotid artery stenosis and peripheral artery disease (PAD), [15], [16] irrespective of diabetes or renal disease.

Atherosclerosis is a generalized disease that develops years before clinical events occur. Previous studies have only focused on symptomatic disease in a single vascular bed (coronary, carotid, or femoral). It is yet unclear whether skin AF is already increased in subjects with subclinical atherosclerosis. We hypothesized that skin AF is increased in patients with subclinical atherosclerosis, independent of diabetes and renal function, and that skin AF is positively associated with the degree of atherosclerosis. Therefore, we compared skin AF in subjects without and with subclinical atherosclerosis as ascertained by non-invasive imaging measures, and in patients with clinically overt and established atherosclerosis.

## Materials and Methods

### Patients

We performed a cross-sectional study of 223 patients, at least 18 years of age, visiting the outpatient vascular clinic of our hospital for primary (*n* = 163) or secondary (*n* = 60) cardiovascular prevention between 2006 and 2012. The study was approved by the local institutional review board at the University Medical Center Groningen and all participants gave written informed consent. Eligible patients of the primary prevention group were referred for counselling because of an increased CVD risk based on conventional cardiovascular risk factors and did not have a history of CVD or symptoms of coronary artery disease, cerebrovascular disease or PAD. The primary prevention group was divided in patients with and without evidence of subclinical atherosclerosis, the latter forming the control group. Subclinical atherosclerosis was defined as the presence of one or more plaques in carotid and femoral arteries using high resolution ultrasonography or a coronary artery calcium score (CACS) >100 on computed tomography (CT). These cut-offs were chosen on the basis of previous reports showing that subjects meeting these criteria are at substantially increased CVD risk. [17], [18] The secondary prevention group consisted of patients with proven PAD, which was ascertained by a resting ankle-brachial index ≤0.90 or a toe-brachial index ≤0.70 if possible in case of non-compressible calf arteries, or a history of radiological or surgical intervention for PAD. PAD was confirmed by CT angiography, magnetic resonance angiography or catheter angiography. For both primary and secondary prevention, exclusion criteria consisted of an estimated glomerular filtration rate of <60 mL/min/1.73 m^2^, a history of renal transplantation, a recent acute coronary syndrome or cerebrovascular attack, or sepsis (all within the past 3 months), cognitive impairment, or current cancer or autoimmune disease because these conditions have previously been shown to increase skin AF. Furthermore, those with a brown or black skin (Fitzpatrick type V skin) were excluded because skin AF could not be reliably measured with the device used in the current study due to excessive light absorption in this skin type. In the primary and secondary prevention group, skin AF and CACS measurements were performed. Since the secondary prevention group of PAD patients by definition had clinically established and proven atherosclerotic disease, no ultrasonographic plaque scoring was performed in this group.

### Risk Factor Assessment

The following risk factors were assessed: smoking status, dyslipidaemia (fasting low density lipoprotein (LDL) cholesterol >4.0 mmol/l, high density lipoprotein (HDL) cholesterol <1.2 mmol/L (female) or HDL-cholesterol <1.0 mmol/L (male), triglycerides >4.0 mmol/L, or current lipid lowering treatment), hypertension (blood pressure ≥140/90 mmHg or drug treatment for hypertension), obesity (body mass index (BMI) ≥30 kg/m^2^), family history of premature CVD (in first degree relatives, male <55 years and female <65 years), and diabetes mellitus (known diabetes, fasting plasma glucose >7.0 mmol/l or random plasma glucose >11.1 mmol/L). Medication use was documented and additional measurements of serum creatinine and high-sensitive C-reactive protein (hsCRP) were performed. Metabolic syndrome was defined according to the International Diabetes Federation, in which BMI substituted waist circumference, because the latter was unavailable. [19] Framingham risk score was defined as the 10 year risk of coronary heart disease (i.e. myocardial infarction or death from coronary heart disease). [20] Kidney function was estimated using the MDRD formula [21].

### Measurement of Skin Autofluorescence

Skin AF was assessed using the AGE Reader (DiagnOptics Technologies BV, Groningen, the Netherlands). This is a non-invasive desk-top device that uses the characteristic fluorescent properties of certain AGEs to estimate the level of AGEs accumulation in the skin. The method has been extensively validated and strongly correlates with individual AGE compounds measured in skin biopsy dermal tissue homogenates taken from the same site as skin AF measurement. [22] Technical details concerning the optical technique have been described elsewhere. [7] In short, the right forearm was positioned on top of the device which is the standard and most practical measuring site for skin AF. The AGE Reader illuminates a skin surface of 4 cm^2^ with an excitation light source with a peak excitation of 370 nm. Emission light (fluorescence in the wavelength of 420–600 nm) and reflected excitation light (with a wavelength of 300–420 nm) from the skin is measured with a spectrometer. Skin AF is calculated as the ratio between the emission light and reflected excitation light, multiplied by 100 and expressed in arbitrary units. A series of three consecutive measurements was carried out, taking less than a minute of time. Mean skin AF was calculated from these three consecutive measurements and used in the analyses. The method is observer independent and has an intra-patient coefficient of variation of 5% [7].

### Plaque Assessment by Intima Media Thickness

Plaque assessment was performed by measurement of carotid and femoral IMT, as described previously. [23] High resolution B-mode ultrasonography (Acuson 128XP10, Acuson Corporation, Mountainview, USA) with a 7 MHz linear array transducer was used with the subject in a supine position. For both carotid arteries, a mean value over 10 mm length of the far wall segments of the common and internal carotid artery and the carotid bulb were imaged from a fixed lateral transducer position. The same procedure was used for the femoral arteries, with measurement of segments of the common femoral artery and the superficial femoral artery. Mean and maximum IMT of each of the 10 segments was calculated. Presence of a plaque was based on the Mannheim consensus statement. A plaque is defined as a focal structure that encroaches onto the arterial lumen at least 0.5 mm or 50% of the surrounding IMT value or demonstrates a thickness of ≥1.5 mm as measured from the media-adventitia interface to the intima-lumen interface. [24] A total plaque score was calculated summing all segments with presence of at least one plaque, consequently ranging from 0 to 10. The sonographers were unaware of the risk factors of the studied persons. The measurements were analyzed offline by an independent image analyst who also was unaware of the clinical status of the patient.

### Measurement of Coronary Calcium Score

CACS was measured either with electron beam CT (C-150 Imatron, San Francisco, USA) or dual-source CT (Siemens Somatom Definition, Forchheim, Germany). A standard scanning protocol was applied. The scan range was from the carina to 1.5 cm below the base of the heart. Images were acquired at 80% of the cardiac cycle for electron beam CT or 70% for dual-source CT, with electrocardiographic triggering, during a single breath-hold. For electron beam CT 38 contiguous, 3 mm thick slices were obtained, with a scan time of 100 ms per slice. Tube voltage was 120 kV, with a tube current of 64 mA and a field-of-view of 260 mm. For dual-source CT a sequential scanning protocol was used, with 6×3 mm collimation, 330 ms rotation time, tube voltage 120 kV, tube current dependent on the weight of the patient and a field-of-view of 250 mm. Quantification of CACS was performed with the use of dedicated software (AccuImage Diagnostics Corporation, South San Francisco, USA for electron beam CT, and Siemens Syngo, Forchheim, Germany for dual-source CT) by trained readers, according to the method described by Agatston. [25] The CACS of the dual-source CT was corrected to correlate better with the values of the reference standard, electron beam CT [26].

### Laboratory Analyses

Venous blood samples were collected into EDTA-containing tubes (1.5 mg/mL). Plasma cholesterol and triglycerides were assayed by routine enzymatic methods (Roche Diagnostics GmbH, Mannheim, Germany). HDL-cholesterol was measured with a homogeneous enzymatic colorimetric test (Roche/Hitachi). HsCRP was determined by nephelometry with a lower limit of 0.175 mg/L (BNII N; Dade Behring, Marburg, Germany). Glucose was measured with an APEC glucose analyzer (APEC Inc., Danvers, MA, USA).

### Statistical Analysis

Based on previous studies in asymptomatic subjects, we expected a standard deviation of 0.3 for skin AF and considered a mean difference of 0.2 arbitrary units (AU) between patients with subclinical and those without subclinical atherosclerosis as clinically relevant. With a power of 80% and an alpha of 0.05 and under the assumption of normality, at least 36 subjects were needed to reject the null hypothesis of no difference in a 2-sided independent t-test.

Normally distributed parameters are shown as mean with standard deviation, non-normally distributed values are given as median (interquartile range) and categorical variables are reported as number (percentage). To investigate differences between the three risk groups, the Kruskal-Wallis test for continuous variables and the Chi-square test for categorical variables was used. In case of a significant difference between the three groups, the Mann-Whitney U test was used to compare the subclinical atherosclerosis group with the control group. Several ordinal logistic regression models were developed to examine the relationship between the atherosclerosis groups and skin AF in addition to other risk factors. In the first model, age, sex, diabetes mellitus and renal function were entered as covariates and in the second model, cardiovascular risk factors defined in the Framingham risk score were added to the first model. In the third model, age, sex, renal function and metabolic syndrome were entered. The odds ratio of increasing level of atherosclerosis (control, subclinical, clinical atherosclerosis) was calculated for each AU increase in skin AF.

The Spearman correlation coefficient was used to examine the univariate association between skin AF and other factors. Those with a *P*<0.10 were included in the linear multiple regression model using stepwise selection of variables. All statistical analyses were performed using PASW Statistics version 18.0.3 (SPSS Inc, Chicago, Illinois, USA). All statistical tests are two-sided. A *P*-value of less than 0.05 was considered statistically significant.

## Results

### Patients

A total of 223 patients participated. Characteristics are shown in Table 1, 2 and 3. In the primary prevention group, 67 subjects had subclinical atherosclerosis, while 96 subjects did not meet our criteria for subclinical atherosclerosis and were assigned to the control group. Age, sex, BMI, systolic blood pressure, fasting glucose, hsCRP, lipids, kidney function, the presence of diabetes mellitus, smoking status, hypertension, and cardiovascular drug use differed significantly between groups. Subjects of the subclinical atherosclerosis group were older, had a higher BMI, systolic blood pressure, and fasting glucose levels as compared with the control group. As expected from their need for secondary prevention, PAD patients used statins more frequently, and consequently had lower lipid levels.

**Table 1:** Clinical characteristics of study groups.

	Controls	Subclinical atherosclerosis	Clinical atherosclerosis	*P-*value
	(*n* = 96)	(*n* = 67)	(*n* = 60)	
Age (years)	43.8±9.5	51.8±7.8	63.5±7.6	<0.001^a^
Sex (Male/Female)	53/43	32/35	44/16	0.01
Skin autofluorescence (AU)	1.87 (1.68–2.12)	2.11 (1.83–2.46)	2.71 (2.15–3.27)	<0.001^a^
Body mass index (kg/m^2^)	25.0 (23.1–27.7)	26.6 (23.8–29.8)	26.3 (24.2–29.5)	0.03^a^
Systolic BP (mmHg)	130 (120–140)	136 (128–146)	139 (127–164)	0.005^a^
Diastolic BP (mmHg)	80 (73–90)	82 (75–90)	80 (75–85)	0.57
Fasting glucose (mmol/L)	5.2 (4.9–5.8)	5.3 (5.1–5.8)	5.5 (5.0–6.4)	0.09
High sensitive CRP (mg/L)	1.20 (0.60–3.00)	1.50 (0.58–3.33)	3.80 (1.35–7.10)	<0.001
Cholesterol (mmol/L)	5.7 (4.7–6.7)	6.0 (4.9–6.7)	4.3 (3.9–5.4)	<0.001
LDL-cholesterol(mmol/L)	3.8 (2.9–4.7)	4.0 (3.3–4.5)	2.5 (2.1–3.2)	<0.001
HDL-cholesterol (mmol/L)	1.3 (1.1–1.7)	1.3 (1.0–1.6)	1.3 (1.1–1.4)	0.35
Triglycerides (mmol/L)	1.36 (0.91–2.10)	1.67 (1.09–2.74)	1.69 (1.12–2.11)	0.12
Serum creatinine (umol/L)	73.5 (68.0–84.0)	70.0 (64.0–76.0)	79.0 (69.0–94.0)	<0.001^a^
GFR	93.7 (83.3–103.8)	96.0 (85.3–105.9)	83.1 (68.3–97.2)	<0.001
Diabetes mellitus	3 (3.1)	2 (3.0)	13 (21.7)	<0.001
Smoking				0.009
Yes	29 (30.2)	27 (40.3)	22 (36.7)	
No	31 (32.3)	17 (25.4)	5 (8.3)	
Past	36 (37.5)	23 (38.8)	33 (55.0)	
Dyslipidaemia	77 (80.2)	59 (88.1)	51 (85.0)	0.39
Hypertension	43 (44.8)	41 (61.2)	52 (86.7)	<0.001^a^
Metabolic syndrome	24 (25.0)	25 (37.3)	21 (35.0)	0.19
Framingham Risk Score	2.0 (0.4–4.1)	5.1 (2.5–11.7)	9.5 (3.2–18.3)	<0.001^a^

^a^ *P*<.05 between subclinical atherosclerosis and controls.

BP = blood pressure; GFR = glomerular filtration rate using MDRD formula.

**Table 2:** Medication use of study groups.

	Controls	Subclinical atherosclerosis	Clinical atherosclerosis	*P-*value
	(*n* = 96)	(*n* = 67)	(*n* = 60)	
Statin use	38 (39.6)	31 (46.3)	46 (76.7)	<0.001
Other lipid lowering medication	8 (8.3)	7 (10.4)	3 (5.0)	0.53
ACEi/ARB use	8 (8.3)	11 (16.4)	37 (61.7)	<0.001
Other antihypertensive medication	14 (14.6)	15 (22.4)	34 (56.7)	<0.001
Aspirin use	4 (4.2)	8 (11.9)	48 (80.0)	<0.001
Coumarin use	0 (0.0)	1 (1.5)	8 (13.3)	<0.001

ACEi = angiotensin-converting enzyme inhibitor; ARB = angiotensin receptor blocker.

**Table 3:** Classification variables for patient group definition.

	Controls	Subclinical atherosclerosis	Clinical atherosclerosis
	(*n* = 96)	(*n* = 67)	(*n* = 60)
Calcium score (AU)	0.0 (0.0–6.9)	130.0 (0.0–248.0)	389.8 (107.6–984.0)
Mean carotid IMT	0.65 (0.57–0.74)	0.83 (0.67–0.98)	NA
Max carotid IMT	0.71 (0.60–0.82)	0.97 (0.80–1.11)	NA
Mean femoral IMT	0.55 (0.48–0.67)	0.70 (0.53–1.05)	NA
Max femoral IMT	0.60 (0.50–0.75)	0.80 (0.63–1.25)	NA

AU = arbitrary units; IMT = intima media thickness.

### Skin Autofluorescence

**Figure 1:**
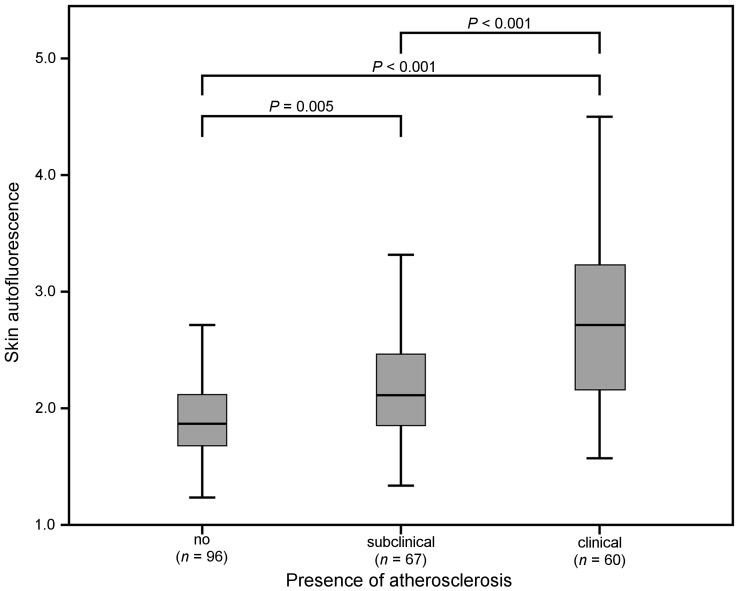
Box plot of skin autofluorescence between patients with increasing degree of atherosclerosis.

Skin AF was significantly higher in patients with subclinical atherosclerosis and PAD compared with controls, and skin AF was higher in the PAD group compared with the group with subclinical atherosclerosis (Figure 1). Within the control group, 40 subjects had no detectable plaques and a CACS of 0 while 56 subjects had only a single plaque and/or CACS of 0–100; skin AF did not differ between these two groups (skin AF 1.93 [1.72–2.16] vs. 1.83 [1.67–2.05], *P* = 0.30). Within the subclinical atherosclerosis group, 17 subjects had plaques only, 10 had an increased CACS only, and 40 had more than one plaque and increased CACS. Between these three groups, skin AF also did not differ (2.14 [1.74–2.45] vs.2.03 [1.80–2.23] vs. 2.16 [1.85–2.55], *P* = 0.47). Finally, in the PAD group, skin AF was not significantly different between those with CACS below and those with CACS above 100 (2.65 [2.10–3.35] vs. 2.72 [2.26–3.20], *P* = 0.92).

The three models for ordinal regression analysis are outlined in Table 4. The difference in skin AF between the 3 groups was independent of age, sex, diabetes, and renal function (*P* = 0.009). Addition of Framingham risk score or replacement of diabetes with metabolic syndrome in this model did not change the results (*P* = 0.017 and *P = 0*.009). In all models, the odds of having a higher degree of atherosclerosis were 2-fold increased per unit of skin AF.

**Table 4:** Odds ratios for increasing degree of atherosclerosis (control, subclinical atherosclerosis, symptomatic peripheral arterial disease) by 1 unit increase of skin autofluorescence (Skin AF).

	Skin AF	
	Odds ratio (95% CI)	*P-*value
Model 1	2.11 (1.21, 3.68)	0.009
Model 2	1.99 (1.13, 3.50)	0.017
Model 3	2.13 (1.21, 3.74)	0.009

Model 1 was only corrected for age, sex, diabetes mellitus and renal function. In model 2, we corrected additionally for cardiovascular risk factors as Framingham risk score. Model 3 was corrected for age, sex, renal function and metabolic syndrome.

To investigate factors associated with skin AF, univariate correlations were calculated in the entire group. Skin AF correlated with age (*r = *0.55; *P*<0.001), Framingham risk score (*r = *0.35; *P*<0.001), diabetes (*r* = 0.23; *P*<0.001), hypertension (*r = *0.19; *P* = 0.005), body mass index (*r = *0.15; *P* = 0.02), CACS (*r* = 0.34; *P*<0.001) and systolic blood pressure (*r* = 0.14; *P* = 0.04), but not with diastolic blood pressure. Skin AF also correlated with plasma glucose (*r* = 0.20; *P* = 0.003), triglycerides (*r* = 0.17; *P* = 0.01), hsCRP (*r* = 0.31; *P*<0.001) and inversely with LDL (*r* = −0.16; *P* = 0.02). No correlations between medication and skin AF were observed. In the linear multiple regression model, skin AF was independently associated with age and plasma glucose (model: *R^2^ = *0.29, *P<*0.001; age: standardized beta 0.46, *P<*0.001; glucose: *r* = 0.19, *P* = 0.005). Because of the low prevalence of diabetes, analyses were repeated after removal of the patients with diabetes. This did not alter the results.

In the primary prevention group, skin AF also correlated with plaque score (*r* = 0.21; *P* = 0.008), mean IMT of carotid and femoral arteries (*r* = 0.22; *P* = .004 and *r* = 0.13; *P* = 0.11) and max IMT (*r* = 0.26; *P* = 0.001 and *r* = 0.21; *P* = 0.007), respectively. The correlation with skin AF and CACS did not reach significance (*r = *0.13; *P = *0.09).

## Discussion

In the current study we demonstrate that skin AF – a non-invasive marker for tissue AGE accumulation – is elevated in subjects with evidence of subclinical atherosclerosis and subjects with clinical atherosclerosis. Furthermore, skin AF increases with the degree of atherosclerosis, independently of factors known to be associated with accumulation of AGEs, including age, sex, diabetes or metabolic syndrome, kidney function and Framingham risk score.

To the best of our knowledge, this is the first study to demonstrate an association of skin AF with varying degrees of atherosclerosis. This was ascertained not only by measuring IMT, but also by plaque assessment and CACS, all of which are validated methods for measuring subclinical atherosclerosis. Earlier, we reported that skin AF is increased and a strong predictor of mortality in diabetes mellitus and end stage renal disease compared with healthy controls. [8], [11] Other groups have confirmed the association between skin AGEs and coronary artery disease in patients with type 1 diabetes, using a different setup, referred to as skin intrinsic fluorescence. [27] The current study is in line with these observations and presents the new finding of a higher accumulation of AGEs in patients with subclinical atherosclerosis, independent of diabetes and renal function, with the simultaneous use of more than one technique [2].

In line with the hypothesis of a role of AGEs beyond diabetes and renal disease, we demonstrated earlier that skin AF is elevated in PAD [15], [16], coronary artery disease [12] and myocardial infarction. [13] Skin AF was shown to correlate with markers of subclinical atherosclerosis, i.e. IMT [14], [28] and small artery elasticity [10], the soluble RAGE and with markers of inflammation, endothelial activation, and oxidative stress in other studies. [12], [13] These previous studies were all performed in populations with known cardiovascular disease, diabetes, renal disease, or autoimmune diseases. This may have confounded the relation between skin AF and atherosclerosis. The diseases per se, especially autoimmune diseases, could cause increases in skin AF. [29] The study by Lutgers [14] demonstrated an association between skin AF and IMT in healthy subjects without diabetes. However, in this particular study plaques were not documented and no coronary calcium score was measured. Also, a preliminary version of the AGE-Reader was used, which has not been developed further and was not validated to skin biopsy.

Correlations between (non-invasive) assessments for different vascular beds i.e. IMT, carotid and coronary plaque scores, and CACS) is moderate, even in post-mortem studies. [30] No single method seems to adequately reflect overall atherosclerosis burden. In line, we found only a weak concordance between the presence of plaques and CACS, with most subjects presenting with plaques or coronary calcifications only. This supports our choice to study multiple vascular techniques and vascular beds. The IMT value is strongly age dependent and a relatively weak predictor of CVD. Therefore, we preferred to define atherosclerosis as the presence of plaques, which is considered a more accurate surrogate for subclinical atherosclerosis [31] and a better predictor of future CVD. [32] Although we used the Mannheim consensus definition, some plaques may have been missed, since we only quantified plaques that were present at the far wall and did not scan outside the designated segments.

CACS is a stronger predictor of future coronary events [33] and to a lesser extent with CVD in general. CACS correlates strongly with histopathologic coronary disease and that absence of calcification is highly suggestive for the absence of CAD. [34] We chose a cutoff of >100 AU for subclinical atherosclerosis, since this is considered optimal to separate asymptomatic subjects at high from those at low risk. Since CACS only detects the presence of a calcified plaque, so-called “soft” plaques are missed. [34] Furthermore, with the techniques we used it is not possible to discriminate “active” or vulnerable from stable plaques. This would necessitate more sophisticated and currently experimental techniques.

Skin AF is an indirect marker for AGEs in the skin, and is influenced by other skin fluorophores. Most AGEs are not fluorescent. Nonetheless, in validation studies, skin AF strongly and consistently correlated with fluorescent as well non-fluorescent AGEs, including the major and most extensively studied AGE N(ε)-carboxymethyl-lysine. [35] We did not collect extra plasma samples and could therefore not assess plasma AGE levels. The disadvantage of plasma AGEs is that they can be influenced and fluctuate due to several factors (i.e. smoking, nutrition, renal function) whereas skin AF remains stable. Furthermore, plasma AGE measurements have an impaired reproducibility due to a lack of uniformity in assays, and are not independently associated with CVD [36].

A restriction to measuring skin AF is that it could not be reliably measured in persons with a dark skin with the AGE-Reader used in this study. However, with the new set-up of the AGE-Reader it is possible to perform accurate measurements in darker skin types, even in Asian or African populations [37].

The use of cardiovascular drugs may have influenced AGE levels. Statins and aspirin have been shown to reduce plasma levels of the soluble receptor for AGEs. [38], [39] Although no such evidence exists for the effects on AGE levels in serum or tissue, this cannot be excluded. If statin use would have caused mitigation of the association between skin AF and extent of atherosclerosis (bias toward zero), then we expect the actual relationship between skin AF and atherosclerosis to be stronger. The majority of patients referred for secondary prevention were treated with lipid and blood pressure lowering drugs according to the latest guidelines, which explains the relatively low levels of cholesterol and blood pressure in these patients.

Since this is a cross-sectional study, a direct etiological role of skin AF or AGEs in atherosclerosis cannot be proven. Furthermore, the relationship between skin AF and severity of atherosclerosis is confounded by the conventional cardiovascular risk factors. Statistical correction inevitably results in interaction and overadjustment, since these factors are strongly interrelated and age dependent. Because of the small study population, we did not correct for all risk factors separately and chose to cluster them in the Framingham risk score and metabolic syndrome. Skin AF should not be considered a diagnostic marker to detect (subclinical) atherosclerosis, but as an additional non-invasive marker for cardiovascular risk.

In conclusion, in this cross-sectional study we confirm the hypothesis that skin AF, a non-invasive marker for AGE accumulation, is elevated in subclinical atherosclerosis and subjects with clinical atherosclerosis. These data add further evidence that accumulation of AGEs is linked to atherosclerosis, even at a subclinical level, independent of age and sex and, importantly, diabetes and kidney disease. However, before applying this method in clinical practice for primary prevention, these results need conformation in a prospective cohort study.
